# Teamwork in home care nursing: A scoping literature review

**DOI:** 10.1111/hsc.13910

**Published:** 2022-07-21

**Authors:** Roger Larsson, Gudbjörg Erlingsdóttir, Johanna Persson, Christofer Rydenfält

**Affiliations:** ^1^ Department of Design Sciences Lund University Lund Sweden

**Keywords:** home care nursing, home care, home healthcare, literature review, nursing, teamwork

## Abstract

Due to an increased number of complex multi‐ and long‐term ill patients, healthcare and nursing provided in patients' homes are expected to grow. Teamwork is important in order to provide effective and safe care. As care becomes more complex, the need for teamwork in home care nursing increases. However, the literature on teamwork in the patients' home environment is limited. The aim of this study is to describe the scope of the current literature on teamwork in home care nursing and outline needs for future research. Seven electronic databases were systematically searched and 798 articles were identified and screened. Seventy articles remained and were assessed for eligibility by two of the authors. Eight themes were identified among the 32 articles that met the inclusion criteria. Studies concerned with teamwork regarding isolated tasks/problems and specific teamwork characteristics were most common. Methods were predominantly qualitative. Multiple method approaches and ethnographic field studies were rare. Descriptions of the context were often lacking. The terms ‘team’ and ‘teamwork’ were inconsistently used and not always defined. However, it is apparent that teamwork is important and home care nurses play a crucial role in the team, acting as the link between professionals, the patient and their families. Future studies need to pay more attention to the context and be more explicit about how the terms team and teamwork are defined and used. More research is also needed regarding necessary team skills, effects of teamwork on the work environment and technology‐mediated teamwork.

**What is known about this topic?**

- Teamwork is associated with improved care quality and increased patient safety.
- Treatment and care performed at home tend to become more complex due to the ageing population, which in turn puts higher demands on care professionals.
- There is a lack of contemporary overviews of existing research regarding teamwork when treating and caring for patients at home.

**What this paper adds?**

- Studies of teamwork in patients' home environments tend to pay too little attention to the context.
- Nurses play a crucial role in teamwork when treating and caring for patients at home.
- More research is needed regarding necessary team skills, effects of teamwork on the work environment and technology‐mediated teamwork.

## 1 INTRODUCTION

This literature review investigates existing knowledge on teamwork in home care nursing. By *home care nursing* we mean treatment and care taking place in the patient's home that is predominantly performed by registered nurses. The definition and organisation of home care vary across countries but can generally be considered any care provided in the patient's home enabling them to stay living in their home environment (Genet et al., 2012). While healthcare and nursing provided in patients' homes are expected to grow in the future, studies regarding teamwork when treating and caring for patients at home are sparse. Thus, it is of considerable interest to describe the current status of the field and to give orientation for future research.

The global population is ageing and the number of people over 65 is projected to more than double by 2050 (United Nations Department of Economic and Social Affairs, Population Division, 2020). As a result, the need for treatment and care in patients' homes is expected to increase. In Sweden, elderly care is expected to grow by 170,000 employees between 2017 and 2035 (Statistics Sweden, 2017), and the expenditure due to shifts from informal to formal care in the EU is expected to more than double between 2013 and 2060 (European Commission, 2015). In the U.S., the need for home health and personal care aides is projected to grow by 34% between 2019 and 2029 (Bureau of Labor Statistics & U.S. Department of Labor, 2021). Elderly patients are already being treated and cared for in their ordinary accommodations, even during multi‐ and long‐term illnesses (The National Board of Health and Welfare, 2014a, 2014b). As a consequence, care becomes increasingly complex and requires that different health care providers and professions cooperate around the patient (Reeves et al., 2010). For example, in Sweden, the expansion of home care is part of a larger effort to trim hospital organisations and give primary care centres and home care nursing increased responsibilities (Stiernstedt et al., 2016). This is not only because caring for patients at home is considered more cost‐effective but also to protect the Western world's prevailing ideals of human autonomy, dignity and identity (Carlander et al., 2009). Making healthcare more mobile and transferring patient treatment and care into the home setting has its advantages and disadvantages. On one hand, elderly people with chronic illnesses have been found to prefer being treated, cared for and even dying at home (Genet et al., 2011; Gomes et al., 2013; Tarricone & Tsouros, 2008). On the other hand, multimorbid patients living at home grade their health lower than those in nursing homes (The National Board of Health and Welfare, 2018). Multimorbid patients are also readmitted more often after being sent home from the hospital (The National Board of Health and Welfare, 2021).

To meet these needs, healthcare must become more effective, and teamwork is perceived to increase effectiveness (Driskell et al., 2018; Reeves et al., 2010). Teamwork in healthcare can be defined as a ‘…dynamic process involving two or more health professionals with complementary backgrounds and skills, sharing common health goals and exercising concerted physical and mental effort in assessing, planning, or evaluating patient care’ (Xyrichis & Ream, 2008, p. 238). Besides organisational effectiveness, teamwork is associated with the delivery of safe and high‐quality care and increased job satisfaction (Kalisch et al., 2010; Lemieux‐Charles & McGuire, 2006; Manser, 2009; Markle‐Reid et al., 2010; Welp & Manser, 2016). At the same time, confusion and inconsistencies exist about the use of team and teamwork terminologies in healthcare (Flores‐Sandoval et al., 2021; Lyubovnikova et al., 2015; Rydenfält, Borell, & Erlingsdottir, 2019).

### 1.1 Aim

The aim of this study is to explore the available literature regarding teamwork in *home care nursing*. More specifically, we intend to describe the scope of the current literature from the perspective of its focus and the methodologies being used and to identify needs for future research.

## 2 MATERIALS AND METHODS

### 2.1 Search strategy

**FIGURE 1:**
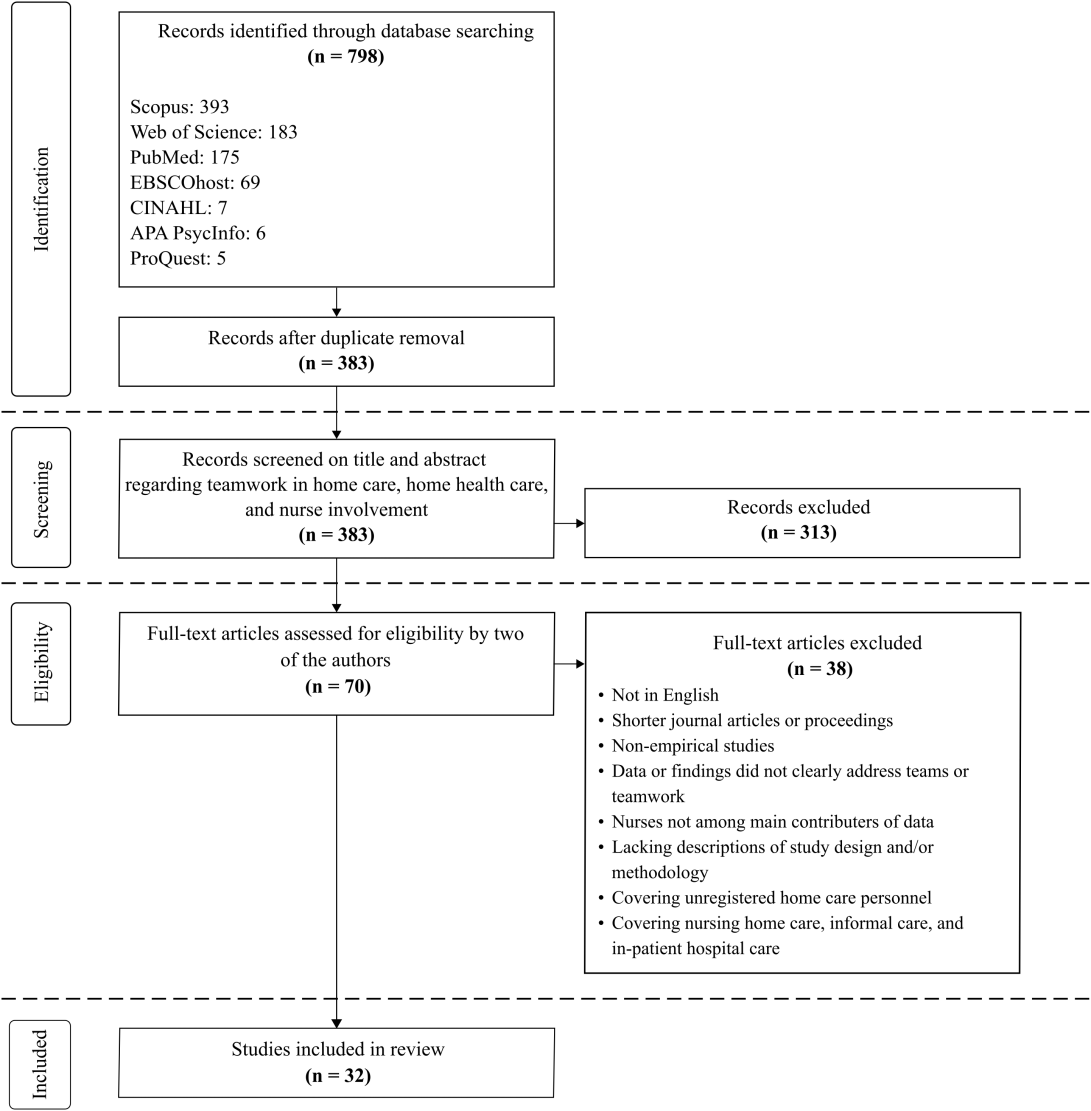
PRISMA flow chart illustrating the data selection process

Review articles can be positioned along a scale from exploratory to explanatory. Compared to systematic reviews of the evidence in relation to specific predefined questions, scoping reviews are usually more explorative, with the intent to ‘…identify and map the available evidence’ (Munn et al., 2018, p. 2). The scoping review design was chosen due to the perceived limited number of studies concerned with teamwork in home care nursing. Related to Arksey and O′Malley's suggested reasons for conducting a scoping review, we focused on ‘the extent, range and nature of research activity’ (Arksey & O'Malley, 2005, p. 21), that is the scope of the current literature, and on ‘research gaps in the existing literature’ (Arksey & O'Malley, 2005, p. 21). The data were collected through literature searches in seven electronic databases: *Scopus*, *Web of Science*, *PubMed*, *ProQuest*, *EBSCOhost*, *CINAHL* and *APA PsycInfo*. The literature search and review processes were inspired by the structured search methodology, *Preferred Reporting Items for Systematic reviews and Meta‐Analyses* (PRISMA) 2009 (Moher et al., 2009). This approach was used to (1) identify, (2) screen, (3) assess and (4) include scientific studies. An overview of the review process is presented in Figure 1.

The data were retrieved from the databases on June 3, 2020 and targeted empirical studies were published in scientific journals between January 1, 2010 and June 1, 2020. Since home care nursing is defined, organised and functions differently around the world, a broad search strategy was used. The search strategy was defined in two levels constrained to the *title*, *abstract* and *keywords* fields. The first level was used to identify possible studies concerning healthcare or care at home and used the following *title* search terms: ‘home care’, ‘home healthcare’, ‘home healthcare’, ‘home care nursing’, ‘home nursing’ and ‘home health services’. The second level used the search term ‘team*’ targeted in the *title*, *abstract* and *keywords* to find studies relating to teamwork. The search string in *Scopus* looked like this:

(TITLE ([“home care” OR “home healthcare” OR “home health care” OR “home care nursing” OR “home nursing” OR “home health service”]) AND TITLE‐ABS‐KEY ([“team*”])) AND DOCTYPE (ar OR re) AND PUBYEAR >2009 AND PUBYEAR <2021.

The broad first level was used due to the difficulties in capturing home care nursing that was contextually similar to that which exists in Sweden. The term team* in the second level also includes studies related to teamwork and was considerably more accurate and straightforward.

### 2.2 Criteria for inclusion and exclusion

The articles included had to focus on teamwork in home care nursing where a team, or an equivalent group of care professionals considered to be a team, had to be present. Team structures were examined and the results related to the data from these were necessary to be included. Articles also had to be empirical studies, written in English, and published as articles in scientific journals. Studies were excluded if their data or findings did not clearly address teams or teamwork, if registered nurses were not main contributors to knowledge (i.e. not part of the team being studied) or if the studies lacked descriptions of study design or methodology. Furthermore, studies covering nursing home care or in‐patient hospital care were excluded as well as studies involving informal home care and basic home care (care provided by aides and assistant nurses).

### 2.3 Data extraction and analysis

Due to the diverse and inconsistent use of terminologies related to teams and teamwork, a qualitative assessment of each study was conducted to determine if they actually were concerned with teamwork (Flores‐Sandoval et al., 2021; Lyubovnikova et al., 2015; Rydenfält et al., 2017; Rydenfält, Borell, & Erlingsdottir, 2019). An inclusive approach to the definition of teams was applied. In practice, this meant that if the identified studies of home care nursing used the terms ‘team’ or ‘teamwork’ or were concerned with factors associated with teamwork in healthcare, they were then included.

The articles remaining after the initial identification and screening stages were split between the first and the fourth authors for full‐text assessments of eligibility. The two authors went through the articles individually on their own. Then they met and discussed their assessments on what studies should be included or excluded and why. This process continued over several iterations until both authors agreed on which studies to include. After this final assessment, the *aim*, *study design*, *subject* and *main results* were described for the remaining articles. Here, ‘subject’ refers to the topic or focus of the assessed study. Then the authors formulated *themes* based on the subjects identified in each study. The classification of themes was based on the subjects and the overall content of each study. Depending on the content, it was possible to categorise some studies under multiple themes. After an initial list of themes had been formulated, the second author joined the discussion to further develop the themes. The analysis was then adjusted and verified by the third author.

## 3 RESULTS

The initial search gave 798 articles. After duplicates were removed, 383 articles remained and were screened. Seventy articles passed the screening. These were then retrieved in full text and assessed for eligibility. The final number of articles left was 32 (see Figure 1). An overview of the 32 studies is presented in Table 1. The majority of the studies originated from Northern and Western Europe, and North America (see Table 2). The articles were distributed over 21 scientific journals. Eight themes were identified in the literature as shown in Table 3. The study designs and quality of the articles varied considerably, from controlled studies to case studies. Qualitative methods dominated: interviews, questionnaires and focus groups were the most commonly used methods (see Table 4). The studies utilised both stand‐alone and multiple methods in their study design; however, the former was most prevalent. When combining methods, the qualitative–qualitative pairing was more common than quantitative–qualitative pairing. Team definitions were not always clearly stated in the literature and sometimes not even present. The studies included did not always clearly describe which professionals constituted the team or which tasks were performed by each professional. However, given the criteria for inclusion, we knew that the teams included had at least one nurse. Descriptions of national and organisational contexts were often given but seldom on a level that provided enough insight into the dynamics of the health care system studied and the organisation of work. The terminology and potential distinction between home healthcare and home care nursing were not always clearly defined either. The results from the included studies across thematic affiliations addressed the need to enhance familiarity and collaboration between team members. The literature also consistently considered teamwork as something good and desirable. We present the eight identified themes in the following sections.

**TABLE 1:** Overview of the 32 articles included in the literature review

Study	Aim	Study design and method	Subject	Main results	Theme
Adekpedjou et al. (2020) (Canada)	To assess home care providers' intentions to engage in interprofessional shared decision‐making in relation to a parent study on the training of interprofessional shared decision‐making	A quantitative questionnaire study	Education and training Intervention Shared decision‐making	Intentions to engage in interprofessional shared decision‐making decreased and their associated factors changed during the study. Discouraged engagement, as well as increased staff workload and turnover, can be a result of a significant increase in extra clients per care provider and major changes in the health and social care systems. Intentions are often associated with moral norms but can also be driven more by practical issues than morally acceptable or desirable ones	Teamwork around a specific task or problem Organisational learning Organisational change
Berggren et al. (2017) (Sweden)	To ‘evaluate, by profession, the effectiveness of an interprofessional educational intervention for district nurses and general practitioners on three areas of nutritional care for patients in a palliative phase’ (Berggren et al., 2017, p. 1)	A quantitative questionnaire study	Education and training Intervention Nutritional care	Significant positive effects were identified for the district nurses and general practitioners in the areas of perceived familiarity and perceived collaboration. For the third area, levels of knowledge, the effects were only significant for general practitioners	Teamwork around a specific task or problem Organisational learning
Bjornsdottir (2018) (Iceland)	To ‘… enhance our knowledge and understanding of the nature of good home care nursing as practice’ (Bjornsdottir, 2018, p. 178)	A qualitative interview and shadowing study	Nature of home care nursing Community home care Facilitate care relations	Nurses perceive good care as dependent on connections and coordination between care participants. Home care nursing is largely a matter of teamwork where responsibilities and expertise are fluid. Values and ideas develop and change over time. It is important to give time to staff engaged in home care to develop a common understanding of each caring situation and authority to decide what needs to be done and when	Descriptive studies of teamwork characteristics Teamwork around a specific task or problem Team skills
Castor et al. (2017) (Sweden)	To ‘explore health care professionals' conceptions of caring for sick children in home care services’ (Castor et al., 2017, p. 2786)	A qualitative focus group study	Paediatric home care	A pre‐requisite for caring for children at home is to give healthcare professionals enough time and flexibility when organising visits, well‐functioning teamwork in home care services and sufficient time for debriefing	Teamwork around a specific patient group
De Groot et al. (2018) (Netherlands)	‘To gain in‐depth knowledge about which aspects home care nurses find attractive about their work’ and ‘to explore whether these aspects vary for home care nurses with different levels of education’ (De Groot et al., 2018, p. 95)	A qualitative online focus group study	Work attractiveness	Nurses find being ‘linchpins’ in the community, having autonomy over care decision‐making, freedom in work schedule, working in self‐directed teams and having a variety of patient situations and nursing activities are important contributors to making work attractive	Teamwork and work environment
Dhollander et al. (2019) (Belgium)	‘To explore the existing barriers to early integration as perceived by the palliative home care teams’ (Dhollander et al., 2019, p. 2)	A qualitative focus group study	Oncology home care	Lacking financial resources, cross‐sectional collaboration, interdisciplinary communication, and existing societal perceptions of palliative care as terminal care counteract early involvement of palliative care for cancer patients at home	Teamwork around a specific patient group
Fløystad et al. (2018) (Norway)	To “…describe aspects of collaboration during interprofessional medication review processes…” (Fløystad et al., 2018, p. 83) and explore how they can strengthen service delivery for elderly home care patients	A qualitative focus group study	Medication management	Team leadership responsibilities for planning and running interprofessional medication reviews as well as involving different professionals are important to create good overviews of elderly home care patients. Professionals need opportunities to meet, communicate, and clarify their respective competencies	Teamwork around a specific task or problem
Fujita et al. (2017) (Japan)	To ‘define the team type by collaboration relationship among’… ‘the three core healthcare professionals’ … ‘doctors, home‐visiting nurses and care managers’ and ‘to clarify the factors that contributed to the successful care by the team types’ (Fujita et al., 2017, p. 1944)	A quantitative questionnaire study	Team types	The following were found to affect collaborative relationships within teams: patient conditions, team members' previous experiences working with each other, doctors' understanding of other professionals, nurses' experience of end‐of‐life care and collaborative practices, training background of care managers as well as the use of communication tools	Descriptive studies of teamwork characteristics
Gonghom and Tantivitayatan (2018) (Thailand)	To ‘propose a model for home health care which provides safety, quality care, and efficiency in the Thai context together with means to assess team collaboration’ (Gonghom & Tantivitayatan, 2018, p. 2)	A digital communication study	Digital communication	Virtual teams in home healthcare are capable of pursuing continuity of care. Technology can help solve problems related to access to services. It can also function as an alternative strategy to address the medical staff shortage. However, the team studied still favoured making medical decisions through synchronous rather than asynchronous communication	Teamwork and digitalisation
Gudnadottir et al. (2019) (Iceland)	To ‘…explore the impact of the integration of home care nursing and social services…’ (Gudnadottir et al., 2019, p. 75). More specifically, they investigate how ‘…home care nursing and social services work together’ (Gudnadottir et al., 2019, p. 75)	A qualitative interview and focus group study	Integrated home care nursing and social service	Despite strong and efficient interdisciplinary coordination between team management in social services and home care nursing, weaknesses in collaboration among care workers were identified. Full integration requires attention to providing members from different groups with opportunities to meet, develop mutual understandings of their specific roles and the care they provide and to create a shared vision. Integration is an active process requiring active leadership to develop	Teamwork around a specific task or problem
Hoff and Scott (2017) (U.S.A.)	To understand “how primary care physicians and staff perceive, experience, and use certain types of patient‐centered medical home work for adapting to new demands…” to gain “…insights into patient‐centered medical home implementation at the workplace level.” (Hoff et al., 2017, p. 226)	A qualitative interview study	Patient‐centered medical home care model	Physicians and health care workers are highly proactive and strategic by nature. They push change forward within organisations irrespective of factors in the work environment such as culture, payment, and leadership if they are provided with positive experiences that reinforce how PCMH activities can be perceived and found to be useful	Teamwork around a specific task or problem
Josefsson and Peltonen (2015) (Sweden)	To “explore district nurses' experiences of working in home care after the transfer of home care to municipals from county councils.” (Josefsson & Peltonen, 2015, p. 2)	A qualitative interview study	Care responsibility transfer	District nurses experience better patient conditions in municipal home care compared to the former county council home care. Simultaneously, their work has become more difficult because of organisational barriers. Municipal organisations do not fully meet the requirements to carry out home care and need to address shortcomings to enable district nurses to work satisfactorily and promote competence development	Organisational change
Karlsson et al. (2015) (Sweden)	To ‘explore home healthcare teams' experiences of pain assessment among care recipients with dementia’ (Karlsson et al., 2015, p. 192)	A qualitative interview study	Pain assessment Dementia home care	Pain assessment in dementia patients is challenging. The assessment is guided by obtaining an understanding of behavioural changes in which team coherence aids the procedure. Complementary experience‐based methods are used and motivated by concerns for ethics and responsibility	Teamwork around a specific task or problem Teamwork around a specific patient group
Klarare et al. (2020) (Sweden)	To ‘describe team leaders' experiences of facilitators and barriers of leadership in specialist palliative home care teams’ (Klarare et al., 2020, p. 104)	A qualitative interview study	Leadership	Team leadership is complex and demanding. It is also challenging in relation to organisational issues, feelings of responsibility and team size. Multilevel demands influence team leaders' vision and leadership, from assignments and leadership tasks to involvement in interpersonal discussions and relationships	Team skills
Klarare et al. (2019) (Sweden)	Identifying team types in Swedish specialist palliative care and exploring connections between the type, maturity and effectiveness in home care teams	A quantitative questionnaire study	Team types Team maturity Team effectiveness	The teams investigated varied predominantly between inter‐ and trans‐professional types. More mature teams tend to work in an integrated manner rather than in parallel, with reportedly higher effectiveness	Descriptive studies of teamwork characteristics
Larsen et al. (2017) (Sweden)	To illustrate how professionals in homemaker services, municipal home care, and hospital‐based care services experience interprofessional collaboration in caring for older people with multimorbidity	A qualitative interview study	Collaboration Geriatric home care	Collaboration across organisations in home health care is complex and influenced by the environment, expectations, roles, and cultures. Simple solutions are rare and instead often based on regulations and structure. Organisational interdependence, close staff interactions, flexibility, and improvisation are identified as key features; not erecting boarders between basic and specialised care	Descriptive studies of teamwork characteristics Teamwork a around specific patient group
Lee et al. (2019) (Australia)	Exploring medication management processes, and describing perceived barriers and challenges by community nurses, pharmacists, and general practitioners providing medication management services for home nursing clients	A qualitative focus group, interview and stakeholder consultation meeting study	Medication management	Client safety, workforce productivity and interprofessional relationships are negatively affected by insufficiencies in interdisciplinary communication, team functionality, organisational or workplace policies, processes and systems. Evidence‐based strategies are needed to improve interdisciplinary medication management and medication safety	Teamwork around a specific task or problem
Légaré et al. (2013) (Canada)	To ‘evaluate healthcare providers' intentions to engage in interprofessional shared decision‐making and to identify factors associated with their intentions’ (Légaré et al., 2013, 215)	A mixed method study based on questionnaires, interviews and a focus group	Shared decision‐making	Home healthcare providers demonstrate positive intentions, but of different underlying factors, to engage in interprofessional decision‐making. It is important to translate interprofessional shared decision‐making into clinical practice for each type of care provider. Lack of time, poor team cohesion and high staff turnover are identified as barriers to avoid	Teamwork around a specific task or problem
Lindblad et al. (2018) (Sweden)	To ‘explore how patient safety is described and addressed in specialised home healthcare from the perspectives of multidisciplinary teams and clinical managers’ (Lindblad et al., 2018, p. 2)	A qualitative interview study	Patient safety	Patient safety in home healthcare is based on a team ideology of enhancing care co‐creation through patient autonomy, competence and relatedness. Efforts to keep patients safe while also improving care are a never‐ending battle where patient behaviour and preferences are in contrast to standardisations and quality assessments	Teamwork around a specific task or problem
Maurits et al. (2017) (Netherlands)	To examine if working in self‐directed teams influences nurses' job satisfaction, assess mediating effects of perceived autonomy over patient care and investigate how education moderates the association between autonomy over patient care and job satisfaction	A quantitative questionnaire study	Job satisfaction	Nurses working in highly self‐directional teams are more satisfied with their jobs. Self‐direction is significantly related to job satisfaction. Autonomy over patient care is positively related to job satisfaction but partly mediated. Registered nurses with bachelor's degrees and certified nursing assistants show a positive relationship between autonomy over patient care and job satisfaction, while registered nurses with associate degrees do not show this significant relationship	Teamwork and work environment
Mertens et al. (2019) (Belgium)	To ‘explore how community nurses experience the collaboration with the general practitioner and the palliative home care team nurse in palliative home care…’ and ‘…the perceived factors influencing this collaboration’ (Mertens et al., 2019, p. 3862)	A qualitative interview study	Collaboration	Team member approachability and acquaintance positively influence collaboration. Community nurses need to be highly adaptable during interprofessional home care collaboration. Doctor–nurse dynamics are still influenced by traditions of old, affecting communication. Specialist palliative home care team nurses' function as experts and mediators when community nurses disagree with general practitioners. Interprofessional education and early socialisation can help improve interprofessional relations and teamwork	Descriptive studies of teamwork characteristics
Nasarwanji et al. (2015) (U.S.A.)	‘To better understand the hospital to skilled home health care transition and workflow challenges…’ (Nasarwanji et al., 2015, p. 187)	A qualitative contextual inquiry and shadowing study	Care transition Geriatric home care	Information access, coordination, communication and teamwork are important factors when transitioning older adult patients from hospital to skilled home healthcare. Skilled home healthcare coordinators need to be able to create referrals using information from the whole team and seamlessly transition the information across healthcare settings	Teamwork around a specific task or problem Teamwork around a specific patient group
Neergaard et al. (2010) (Denmark)	To describe experiences and views of health professionals on interprofessional collaboration in basic‐level palliative home care	A qualitative group interview study	Cooperation	Problems are indicated at both the organisation level and among health professionals regarding work culture. The main issues to improve care delivery are task distribution, information exchange, availability, respect and personal acquaintance within the team	Descriptive studies of teamwork characteristics
Noguchi‐Watanabe et al. (2019) (Japan)	To evaluate long‐term ‘…effects of an interprofessional collaboration promotion program among community healthcare professionals’ (Noguchi‐Watanabe et al., 2019, p. 660)	A quantitative questionnaire study	Collaboration Education and training Intervention	Interprofessional collaboration, familiarity and meeting and talking improved significantly for those attending the program. Improvements were not significantly different between one‐ and two‐time workshop participants	Descriptive studies of teamwork characteristics Organisational learning
Perron et al. (2019) (Switzerland)	To explore home care professionals' practices and perceptions regarding written interprofessional communication	A qualitative communication and focus group study	Written communication skills	Interprofessional written communication is rarely explicit. Health professionals write about different topics based on their profession. A lack of clarity exists about what to document and for whom. Openly accessible notebooks at home make health professionals unsure of how to manage confidential information sharing among themselves vis‐à‐vis the desire to actively involve patients and families	Team skills
Pype et al. (2015) (Belgium)	To describe ‘the evaluation of a training program for palliative home care team nurses to act as facilitators of general practitioners' workplace learning’ (Pype et al., 2015, p. 459)	A mixed method interview, progress report and video recording study	Education and training Intervention Workplace learning	A feasible but complex intervention is to train palliative home care team nurses to facilitate general practitioners' workplace learning. Careful and individualised mentoring is required where personal characteristics, interpersonal relationships and contextual variables need to be accounted for	Organisational change Organisational learning
Pype et al. (2014) (Belgium)	The study aims ‘…to identify what, how and from whom health care professionals learn…’ (Pype et al., 2014, p. 1) during interprofessional collaboration in palliative home care	A quantitative questionnaire study	Collaboration Education and training Workplace learning	General practitioners and palliative home care team nurses learn a lot from collaboration in primary palliative care. Learning activities, content and from whom they learn to depend on their profession. General practitioners learn equally from the discussion, reflection, listening and observation, whereas nurses learn more from listening and observation. Learning from discussion and reflection is less prevalent for nurses even though they share expertise with professionals and non‐professionals alike	Descriptive studies of teamwork characteristics Organisational learning
Ree and Wiig (2019) (Norway)	To explore ‘Employees' perceptions of patient safety culture in Norwegian nursing homes and home care services (title), and to assess how cultural dimensions contribute to the overall perceptions of patient safety	A quantitative questionnaire study	Patient safety	The investigated nursing homes and home care services had higher incident reporting and non‐punitive mistake responses than other international studies. Even though perceived differently, both view staffing and teamwork as being important to patient safety. Mutual trust and collaboration in home care teams as well as enabling open communication and value assurance about ideas and suggestions given in nursing homes are considered important to improve patient safety	Teamwork around a specific task or problem
Shaw et al. (2016) (Canada)	To explore ‘…views of an interprofessional group of home care providers…regarding a pilot project encouraging’ (Shaw et al., 2016, p. 262) interprofessional teamwork in palliative home care services	A qualitative interview study	Education and training Intervention	Three key strategies are identified to maximise interprofessional palliative care: giving time and space for service providers to share experiences and foster common goals, establishing a clear team leader that emphasises sharing power among team members and encouraging mutual emotional support during team interactions	Organisational change Organisational learning
Sun et al. (2019) (Canada)	To ‘explore the barriers and enablers of deprescribing from the perspectives of home care nurses, as well as to conduct a scalability assessment of an educational plan to address the learning needs of home care nurses about deprescribing’ (Sun et al., 2019, p. 2)	A qualitative focus group study	Medication management Patient safety	Safe polypharmacy management and deprescription challenges for older home care patients are associated with a lack of open communication, inconsistent medication reconciliation practices, inadequate partnerships and ineffective interprofessional healthcare provider collaboration. Home care nurses have a vital role in medication management and polypharmacy reduction, emphasising the need for more consistent and standardised approaches in educating healthcare providers, informal caregivers and patients alike	Teamwork around a specific task or problem
Tourangeau et al. (2014) (Canada)	‘To identify factors affecting Canadian home care nurse intention to remain employed’ (Tourangeau et al., 2014, p. 1015)	A qualitative focus group study	Staff retention	Job satisfaction is not shown to affect home care nurses' intentions to remain employed. Factors of autonomy; flexibility; workload; supportive work relationships with patients, families, nursing colleagues and supervisors; opportunities for orientation and education; personal safety; travel demands; clinical support and physical resource availability; employment status and pay were found to be influential	Teamwork and work environment
Vaartio‐Rajalin et al. (2019) (Finland)	To ‘describe nurses' experiences of working in home healthcare and their suggestions for the development of this public healthcare sector’ (Vaartio‐Rajalin et al., 2019, p. 260)	A qualitative interview study	Experiences in daily work	Affirmative work shifts for nurses go smoothly and as planned without unexpected tasks, with enough time to document and discuss clients. Non‐affirmative work shifts were comprised of barriers to manage tasks smoothly, burdensome IT programs and an unclear division of responsibility. The more that nurses are engaged in patient‐related nursing activities, patient‐centeredness, collaboration and forward planning, the more perceived influence they have over their work. Suggestions for improvement include collaboration between settings, revised task division, revised IT resources and flexible schedule planning	Descriptive studies of teamwork characteristics

**TABLE 2:** Regions and countries of the included literature

Regions	Countries	Number of studies
Northern Europe		14
	Sweden (Berggren et al., 2017; Castor et al., 2017; Josefsson & Peltonen, 2015; Karlsson et al., 2015; Klarare et al., 2019; Klarare et al., 2020; Larsen et al., 2017; Lindblad et al., 2018)	8
	Iceland (Bjornsdottir, 2018; Gudnadottir et al., 2019)	2
	Norway (Fløystad et al., 2018; Ree & Wiig, 2019)	2
	Denmark (Neergaard et al., 2010)	1
	Finland (Vaartio‐Rajalin et al., 2019)	1
Western Europe		7
	Belgium (Dhollander et al., 2019; Mertens et al., 2019; Pype et al., 2014; Pype et al., 2015)	4
	Netherlands (De Groot et al., 2018; Maurits et al., 2017)	2
	Switzerland (Perron et al., 2019)	1
North America		7
	Canada (Adekpedjou et al., 2020; Légaré et al., 2013; Shaw et al., 2016; Sun et al., 2019; Tourangeau et al., 2014)	5
	U.S.A. (Hoff & Scott, 2017; Nasarwanji et al., 2015)	2
Eastern Asia		3
	Japan (Fujita et al., 2017; Noguchi‐Watanabe et al., 2019)	2
Southeast Asia		1
	Thailand (Gonghom & Tantivitayatan, 2018)	1
Australia and New Zealand		1
	Australia (Lee et al., 2019)	1
Total		32

**TABLE 3:** Number of times the identified themes occurred in the included literature

Themes	Number of studies
Teamwork around a specific task or problem (Adekpedjou et al., 2020; Berggren et al., 2017; Bjornsdottir, 2018; Fløystad et al., 2018; Gudnadottir et al., 2019; Hoff & Scott, 2017; Karlsson et al., 2015; Lee et al., 2019; Légaré et al., 2013; Lindblad et al., 2018; Nasarwanji et al., 2015; Ree & Wiig, 2019; Sun et al., 2019)	13
Descriptive studies of teamwork characteristics (Bjornsdottir, 2018; Fujita et al., 2017; Klarare et al., 2019; Larsen et al., 2017; Mertens et al., 2019; Neergaard et al., 2010; Noguchi‐Watanabe et al., 2019; Pype et al., 2014; Vaartio‐Rajalin et al., 2019)	9
Organisational learning (Adekpedjou et al., 2020; Berggren et al., 2017; Noguchi‐Watanabe et al., 2019; Pype et al., 2014; Pype et al., 2015; Shaw et al., 2016)	6
Teamwork around a specific patient group (Castor et al., 2017; Dhollander et al., 2019; Karlsson et al., 2015; Larsen et al., 2017; Nasarwanji et al., 2015)	5
Organisational change (Adekpedjou et al., 2020; Josefsson & Peltonen, 2015; Pype et al., 2015; Shaw et al., 2016)	4
Team skills (Bjornsdottir, 2018; Klarare et al., 2020; Perron et al., 2019)	3
Teamwork and work environment (De Groot et al., 2018; Maurits et al., 2017; Tourangeau et al., 2014)	3
Teamwork and digitalisation (Gonghom & Tantivitayatan, 2018)	1

**TABLE 4:** Combinations of methods used in the included literature

Methods	Number of studies
Single method (Adekpedjou et al., 2020; Berggren et al., 2017; Castor et al., 2017; De Groot et al., 2018; Dhollander et al., 2019; Fløystad et al., 2018; Fujita et al., 2017; Gonghom & Tantivitayatan, 2018; Hoff & Scott, 2017; Josefsson & Peltonen, 2015; Karlsson et al., 2015; Klarare et al., 2019; Klarare et al., 2020; Larsen et al., 2017; Lindblad et al., 2018; Maurits et al., 2017; Mertens et al., 2019; Neergaard et al., 2010; Noguchi‐Watanabe et al., 2019; Pype et al., 2014; Ree & Wiig, 2019; Shaw et al., 2016; Sun et al., 2019; Tourangeau et al., 2014; Vaartio‐Rajalin et al., 2019)	25
Interviews (Hoff & Scott, 2017; Josefsson & Peltonen, 2015; Karlsson et al., 2015; Klarare et al., 2020; Larsen et al., 2017; Lindblad et al., 2018; Mertens et al., 2019; Shaw et al., 2016; Vaartio‐Rajalin et al., 2019)	9
Questionnaires (Adekpedjou et al., 2020; Berggren et al., 2017; Fujita et al., 2017; Klarare et al., 2019; Maurits et al., 2017; Noguchi‐Watanabe et al., 2019; Pype et al., 2014; Ree & Wiig, 2019)	8
Focus groups (Castor et al., 2017; De Groot et al., 2018; Dhollander et al., 2019; Fløystad et al., 2018; Sun et al., 2019; Tourangeau et al., 2014)	6
Group interviews (Neergaard et al., 2010)	1
Communication studies (Gonghom & Tantivitayatan, 2018)	1
Multiple methods (Bjornsdottir, 2018; Gudnadottir et al., 2019; Lee et al., 2019; Légaré et al., 2013; Nasarwanji et al., 2015; Perron et al., 2019; Pype et al., 2015)	7
Communication studies and focus groups (Perron et al., 2019)	1
Contextual inquiry and shadowing (Nasarwanji et al., 2015)	1
Focus groups and interviews (Gudnadottir et al., 2019)	1
Focus groups, interviews and stakeholder consultation meetings (Lee et al., 2019)	1
Focus groups, questionnaire and interviews (Légaré et al., 2013)	1
Interviews, progress reports and video recordings (Pype et al., 2015)	1
Interviews and shadowing (Bjornsdottir, 2018)	1
Total	32

### 3.1 Teamwork around specific patient groups

This theme consists of studies where teamwork is used to care for specific patient groups including geriatric home care (Larsen et al., 2017; Nasarwanji et al., 2015), paediatric home care (Castor et al., 2017), oncology home care (Dhollander et al., 2019) and dementia home care (Karlsson et al., 2015). Castor et al. (2017) describe the importance of teamwork when caring for sick children at home. Dhollander et al. (2019) show that early involvement of palliative home care efforts can be counteracted in part by ineffective teamwork. The importance of team coherence when conducting pain assessments in dementia patients is highlighted by Karlsson et al. (2015). Larsen et al. (2017) state that professionals in community home care teams need to interact closely, flexibly and collaborate interdependently across organisational boarders to care for multimorbid elderly patients at home. When transferring older patients from hospitals to home health care, Nasarwanji et al. (2015) find that care coordinators play an important role since they assemble collective information from teams and transfer it across healthcare boundaries. This theme highlights the importance of teamwork, often multidisciplinary, when caring for patient groups that suffer from complex multimorbidity.

### 3.2 Teamwork around a specific task or problem

This theme includes studies that describe how teamwork is used to target specific tasks or problems like medication management (Fløystad et al., 2018; Lee et al., 2019; Sun et al., 2019), patient safety (Lindblad et al., 2018; Ree & Wiig, 2019; Sun et al., 2019), shared decision‐making (Adekpedjou et al., 2020; Légaré et al., 2013), nutritional care (Berggren et al., 2017), community home care (Bjornsdottir, 2018), integrated home care nursing and social service (Gudnadottir et al., 2019), patient centred medical home (PCMH) care (Hoff & Scott, 2017), pain assessment (Karlsson et al., 2015) or patient care transition (Nasarwanji et al., 2015). Both Lee et al. (2019) and Sun et al. (2019) report on how inadequate interdisciplinary and interprofessional teamwork among members negatively affects patient medications. Fløystad et al. (2018) identify team leadership to be important in both planning and running medication reviews. All three studies acknowledge that home care nurses have a vital role in the teamwork surrounding medication management. Lindblad et al. (2018) consider the team ideology of co‐creation between patient and home healthcare professionals to be important when confronted with conflicting demands between patients' preferences and quality‐ and safety‐related standards. Furthermore, Ree and Wiig (2019) show that teamwork is the strongest predictor for overall perceived patient safety in home care services. Légaré et al. (2013) and Gudnadottir et al. (2019) both highlight the importance of establishing stable and coherent teams across professional and organisational boarders in order to handle shared problems. Healthcare professionals are overall positive about engaging in interprofessional shared decision‐making, but sometimes are hindered from doing so (Légaré et al., 2013), and interdependent organisations with seemingly fully integrated operations can have more coherency at the top than at the shop floor level (Gudnadottir et al., 2019). Hoff and Scott (2017) illustrate how physicians and healthcare workers engage in team‐based care activities in accordance with the PCMH model (American College of Physicians, n.d.) often applied in the U.S. context, to manage tasks present in their daily work. The studies on this theme are concerned with teamwork around a specific task or problem. A common observation is that there is a considerable focus on the task or problem itself, but less attention is given to the wider context of what teamwork contributes to the practice. It is also clear that nurses often play a central role in these types of teams.

### 3.3 Organisational learning

This theme includes studies describing teamwork improvement efforts in an organisation, either through workplace learning (Pype et al., 2014, 2015) or professional education and training (Adekpedjou et al., 2020; Berggren et al., 2017; Noguchi‐Watanabe et al., 2019; Pype et al., 2014, 2015; Shaw et al., 2016). Shaw et al. (2016) state that team members need time and space for sharing experience, a clear team leadership emphasising power sharing, and mutual emotional support to encourage interprofessional teamwork in palliative home care. In two studies, Pype et al., 2014, 2015 investigate relational dynamics between nurses and general practitioners in palliative home care. Their first study finds that general practitioners are engaged in both transmitting and receiving forms of workplace learning, while nurses predominantly are the receiving actors (Pype et al., 2014). The second study indicates that when nurses are trained to act as facilitators for general practitioners' workplace learning, the outcomes show that it is possible but complex and should be handled carefully through an individualised approach (Pype et al., 2015). Berggren et al. (2017) and Noguchi‐Watanabe et al. (2019) both report on the significant positive effects of interprofessional education and promotion programs.

### 3.4 Organisational change

This theme includes studies that describe changes in organisations that affect teamwork (Adekpedjou et al., 2020; Josefsson & Peltonen, 2015; Pype et al., 2015; Shaw et al., 2016). According to Adekpedjou et al. (2020), intentions to engage in shared interprofessional decision‐making among home care teams decrease during major organisational restructuring events. Josefsson and Peltonen (2015) report on accounts from home care district nurses of better patient conditions after a nationwide care responsibility transfer from county councils to municipalities. The improvements came, however, at the cost of their own work situation. What the studies in this theme have in common is that they are concerned with the improvement of teamwork on a more general level than in relation to specific tasks. However, they also show that this type of organisational change was not always easy to implement or successful (Adekpedjou et al., 2020; Josefsson & Peltonen, 2015).

### 3.5 Descriptive studies of teamwork characteristics

This theme encompasses studies of assessment of team types (Fujita et al., 2017; Klarare et al., 2019), team maturity (Klarare et al., 2019), team effectiveness (Klarare et al., 2019), collaboration (Larsen et al., 2017; Mertens et al., 2019; Noguchi‐Watanabe et al., 2019; Pype et al., 2014), cooperation (Neergaard et al., 2010), experiences in daily work (Vaartio‐Rajalin et al., 2019) and the nature of home care nursing that includes teamwork (Bjornsdottir, 2018). Bjornsdottir (2018) deems home care nursing to largely be about teamwork with fluid expertise and responsibilities, where values and ideas change over time. Nurses perceive that good care depends on connections and coordination among the community care participants surrounding the patient. Fujita et al. (2017) and Klarare et al. (2019) both explore aspects of team types in practice. Fujita et al. (2017) find collaborative relationships within teams to be affected by factors such as team members' experiences of working together, use of communication tools, doctors' understanding of other professionals, nurses' previous experiences of end‐of‐life care and collaborative practice and the training background of care managers. Klarare et al. (2019) find mature teams to be better integrated and to work more effectively than less mature teams that work more in parallel. Neergaard et al. (2010) identify problems at both the organisational and professional levels in basic‐level palliative home care, where the main issues concerned task distribution, information exchange, availability, mutual respect and personal acquaintance among team members. Mertens et al. (2019) find collaboration among community nurses, general practitioners and team nurses who are specialists in palliative home care to be positively influenced by approachability and acquaintance between team members.

The studies on this theme emphasise the importance of networking, coordination and integration to achieve better cooperation and collaboration. Overall, few of the studies applied methods suitable for capturing the complexity of teamwork in context. An exception is Bjornsdottir's (2018) ethnographic study. The remaining were single‐method studies relying on questionnaires or interviews (see Table 4).

### 3.6 Team skills

This theme includes studies where skills considered important for teamwork are described, examples being facilitating care relations (Bjornsdottir, 2018), leadership (Klarare et al., 2020) and written communication skills (Perron et al., 2019). Klarare et al. (2020) find team leadership in specialist palliative home care to be complex and challenging. Demands and personal feelings influence the leader's vision and leadership, from assignments and leadership tasks to interpersonal discussions and relationships. Perron et al. (2019) show that written interprofessional communication lacks clarity, and that uncertainty exists among health professionals about what to document and for whom.

### 3.7 Teamwork and work environment

The studies on this theme describe the relationship between teamwork and factors associated with the work environment such as job satisfaction (Maurits et al., 2017), staff retention (Tourangeau et al., 2014) and work attractiveness (De Groot et al., 2018). Additionally, De Groot et al. (2018) state that nurses find their role as linchpins and leading professionals in the community to be of importance to their perceived work attractiveness. Maurits et al. (2017) find nurses' experiences of working in self‐directed teams to be positively related to job satisfaction and partly mediated by autonomy over patient care. Tourangeau et al. (2014) report that nurses' intentions to remain employed are influenced, among other things, by their relationships with patients, families, nursing colleagues and supervisors. All studies on this theme highlight the importance of autonomy and self‐direction to home care nurses.

### 3.8 Teamwork and digitalisation

This theme consists of one study that examines the attempt to support teamwork through digital means (Gonghom & Tantivitayatan, 2018). These authors investigate a proposed model for virtual team collaboration in home healthcare where team communication and collaboration are mediated through technology. They conclude that technology can help solve problems related to service accessibility and even be a possible strategy to address medical staff shortages. However, healthcare workers were found to favour synchronous over asynchronous communication during consultations regarding medical decision‐making.

## 4 DISCUSSION

This study reviews the scope of the existing literature on teamwork in home care nursing. As previous studies have indicated, the terms team and teamwork are not always used in a consistent manner (Lyubovnikova et al., 2015; Rydenfält, Borell, & Erlingsdottir, 2019; West & Lyubovnikova, 2012). In comparison, the included studies did not always give a good indication of which professionals constituted the team (e.g. Adekpedjou et al., 2020; Dhollander, 2019; Larsen et al., 2017) or of the overall organisational context dynamics (e.g. Lee et al., 2019; Nasarwanji et al., 2015; Noguchi‐Watanabe et al., 2019). This is problematic since subtle differences in team organisation and conceptualization can have significant implications for how the practice functions. In addition, it is not uncommon for study results to include teamwork as only one aspect while also investigating or even targeting something else (e.g. Castor et al., 2017; De Groot et al., 2018; Lee et al., 2019; Ree & Wiig, 2019; Sun et al., 2019). As in healthcare in general, the included studies frame teamwork as something positive (Reeves et al., 2010), and thus, the utility of teamwork is seldom critically discussed.

In the following sections, we will discuss the implications of the results. In turn, we will address *a need for richer descriptions of the context of teamwork*, *the relational nature of teamwork in home care nursing*, that *home care nurses tie the team together*, *teamwork as a source of control and support at work* and that *digitalisation of home care nursing in relation to teamwork is under‐researched*.

### 4.1 A need for richer descriptions of the context of teamwork

The emphasis of the literature is manifested through the most common theme: the use of *teamwork around a specific task or problem*. This is natural since teams can play an important role in the organisation's ability to manage tasks related to care. However, teamwork constitutes and affects more than the handling of specific isolated tasks or problems, such as patient safety and medication management. An analysis that is too narrow leaves out much of the context and risks missing aspects that are important in order to understand teamwork. For example, the staff's perspectives, including personal growth and contributions to the work environment, do not come to the fore in the literature, except for the small theme: *teamwork and the work environment*. These aspects are associated with engagement in efficient and effective teamwork (Hackman, 2002). As with the largest theme (*teamwork around a specific task or problem*), the studies in the second largest theme, *descriptive studies of teamwork characteristics*, also tended to give too little attention to the context. This indicates that there is an overall need to provide richer descriptions of contextual settings when studying teamwork in home care nursing. These descriptions should include a more theoretically driven analysis and the use of methods better suited to capture the nuances and complexity of the studied context. Holistic approaches that apply method triangulation and thick case descriptions (Flyvbjerg, 2006; Geertz, 1973) are largely missing, although there are some examples to the contrary, such as Bjornsdottir's (2018) study. Thus, there is a need for more ethnographic studies and studies that mix complementary methods in order to capture the broader context and provide a more complete picture of the object being investigated. Ultimately, this is important to ensure the generalizability and transferability of the results. A thick description helps the reader to relate the study findings to other cases and determine their applicability (Firestone, 1993).

### 4.2 The relational nature of teamwork in home care nursing

Several studies elevate the importance of familiarity and collaboration between team members (e.g. Berggren et al., 2017; Larsen et al., 2017; Noguchi‐Watanabe et al., 2019; Pype et al., 2014; Ree & Wiig, 2019). Along with the allocation of time and resources, organisations need to loosen their boarders to allow fluid collaboration and interaction to take place between the professionals, enabling them to do what must be done. Caring for patients at home is a complex matter in a complex environment where cases need to be tailored during planning, execution and evaluation. As team members do not necessarily come from the same unit and may thus not be aware of one another's prerequisites beforehand, the team must be allowed to create a common understanding of the whole situation and the team member's individual roles and contributions. Thus, it is important for team members to have past experience of working together and to be acquainted with each other (e.g. Fujita et al., 2017; Mertens et al., 2019; Neergaard et al., 2010). By becoming familiar with each other, team members can start to share experiences and information, build relationships and trust and create shared goals and coherency within the team. These results are consistent with other theories on teamwork in healthcare (Rydenfält et al., 2017). This creates opportunities for *organisational learning*, which can be further enforced through appropriate *organisational change* and interpersonal *team skills*. However, interventions to improve teamwork need to be carefully considered beforehand and executed in relation to the staff's situation. Otherwise, they may affect teamwork negatively and increase employee turnover.

### 4.3 Home care nurses tie the team together

Several studies highlighted that home care nurses fill a critical role in the team when treating and caring for patients at home (Bjornsdottir, 2018; Fløystad et al., 2018; Lee et al., 2019; Sun et al., 2019). On several occasions, the nurses are portrayed as being situated at the core of teamwork, and as the glue that holds care together. Nurses act as a link between the patient and other professionals, thus mantling the role of being responsible for the coordination of care on behalf of the patients and their families. In contrast, according to the PCMH model used in the U.S. (Hoff & Scott, 2017), primary care physicians are pointed out as being the care coordinators (American College of Physicians, 2021). Several studies include the interaction between nurses and general practitioners in their description of teamwork, either in part or as the main focus (Berggren et al., 2017; Fujita et al., 2017; Mertens et al., 2019; Pype et al., 2014, 2015). This is natural because of the incorporated hierarchical dependence nurses experience with physicians. However, in home care nursing, nurses are often more independent. Since physicians may be absent or hard to reach for consultation during home visits, for example nurses need to act as leaders of their teams (De Groot et al., 2018; Josefsson & Peltonen, 2015), a characteristic otherwise often associated with physicians in other healthcare settings.

### 4.4 Teamwork as a source of control and support at work

The *teamwork and the work environment* theme indicate that home care nurses want control over their work situation. This is in line with the Job‐Demand‐Control‐Support model (Karasek, 1990). As demands increase, so does the need for control. The nurses' need for control is reflected by the emphasis on autonomy, self‐direction, flexibility and authority in the results (e.g. Bjornsdottir, 2018; De Groot et al., 2018; Larsen et al., 2017; Lindblad et al., 2018; Maurits et al., 2017; Tourangeau et al., 2014). Effective and well‐functioning teamwork can also act as a source of support to individual nurses in terms of expertise and emotional support among fellow team members in intra‐ and interprofessional constellations (San Martín‐Rodríguez et al., 2005; Shaw et al., 2016; Tourangeau et al., 2014).

### 4.5 Digitalisation of home care nursing in relation to teamwork is under‐researched

Most striking is the lack of literature on *teamwork and digitalisation* in home care nursing. There is a strong ongoing trend of digitalization and digitisation in society. This trend is also evident in home care and home care nursing (Frennert, 2021; Rydenfält et al., 2021; Rydenfält, Persson, et al., 2019). But compared with the advanced technological equipment frequently found in hospital settings, home care is often digitalized and digitised with everyday, off‐the‐shelf technology. When nursing is performed outside institutional settings, assets for communication and documentation of work become essential. As care becomes more mobile, technology has an immense potential to both mediate and improve teamwork.

### 4.6 Limitations

As the definitions and organisation of home care and home healthcare vary between countries and contexts, it is possible that we have missed studies because of the variance in terminology even though the search strategy applied was very inclusive. Twenty‐one out of the 32 included studies originate from Northern and Western Europe. This could have to do with how the term ‘home care nursing’ is defined by us as researchers, and how it is contextually manifested in the Scandinavian countries. Scandinavian home care nursing is broadly implemented, thus making it reasonable for countries with less‐developed home care nursing and home healthcare to be able to learn from how the Scandinavian ones are organised. Some of the identified themes are closely related, for example *Teamwork around a specific patient group* and *Teamwork around a specific task or problem*, as well as *Descriptive studies of teamwork characteristics* and *Team skills*. However, these studies have been classified as distinct enough to be placed in separate themes.

## 5 CONCLUSIONS

Teamwork in home care nursing is a limited research field, constituted of studies within a broad scope. The methods used are predominantly qualitative with a single‐data collection method. The studies often target teamwork as a means to manage specific tasks or problems. However, many studies lack depth when it comes to the description of the context of teamwork. Thus, future studies need to apply methods better capable of capturing the context, for example ethnographic methods. It is apparent that teamwork in home care nursing is dependent on relationships within the team, and that the home care nurse plays a central role in the management and sustainment of those relationships. Furthermore, teamwork is seldom theorised in the literature, merely described. More research is needed overall, but especially regarding important team skills, how teamwork affects the work environment, and digitalisation in relation to teamwork in home care nursing.

## AUTHOR CONTRIBUTIONS

The authors conceptualised the review together. RL and CR were mainly responsible for data extraction and analysis. GE and JP assisted with the analysis. RL was main responsible for drafting the manuscript. However, all authors contributed to the writing.

## CONFLICT OF INTEREST

None.

## Data Availability

Since the article is a review, we have not collected any original data. However, the references to the articles that the review is based on are provided in the manuscript.
